# Efficiency Evaluation of Food Waste Materials for the Removal of Metals and Metalloids from Complex Multi-Element Solutions

**DOI:** 10.3390/ma11030334

**Published:** 2018-02-26

**Authors:** Lorenzo Massimi, Antonella Giuliano, Maria Luisa Astolfi, Rossana Congedo, Andrea Masotti, Silvia Canepari

**Affiliations:** 1 Department of Chemistry, Sapienza University of Rome, Piazzale Aldo Moro, 5, 00185 Roma, Italy; antonella.giuliano@uniroma1.it (A.G.); marialuisa.astolfi@uniroma1.it (M.L.A.); silvia.canepari@uniroma1.it (S.C.); 2 Istituto di Istruzione Superiore “Quinto Ennio”, Corso Roma, 100, 73014 Gallipoli, Italy; mariellaine@tiscalinet.it; 3 Children’s Hospital Bambino Gesù—IRCCS, Research Laboratories, Viale San Paolo, 15, 00145 Rome, Italy; andrea.masotti@opbg.net

**Keywords:** low-cost materials, food waste adsorbents, biosorption, adsorption capacities, elements’ removal efficiency, metals, heavy metal wastewater, environmental remediation, adsorbent surfaces, adsorbents’ chemical structures

## Abstract

Recent studies have shown the potential of food waste materials as low cost adsorbents for the removal of heavy metals and toxic elements from wastewater. However, the adsorption experiments have been performed in heterogeneous conditions, consequently it is difficult to compare the efficiency of the individual adsorbents. In this study, the adsorption capacities of 12 food waste materials were evaluated by comparing the adsorbents’ efficiency for the removal of 23 elements from complex multi-element solutions, maintaining homogeneous experimental conditions. The examined materials resulted to be extremely efficient for the adsorption of many elements from synthetic multi-element solutions as well as from a heavy metal wastewater. The 12 adsorbent surfaces were analyzed by Fourier transform infrared spectroscopy and showed different types and amounts of functional groups, which demonstrated to act as adsorption active sites for various elements. By multivariate statistical computations of the obtained data, the 12 food waste materials were grouped in five clusters characterized by different elements’ removal efficiency which resulted to be in correlation with the specific adsorbents’ chemical structures. Banana peel, watermelon peel and grape waste resulted the least selective and the most efficient food waste materials for the removal of most of the elements.

## 1. Introduction

Today, excessive release of wastewater particularly rich in heavy metals from industrial activities is a critical environmental problem worldwide [1]. Many metals and metalloids, which are toxic and carcinogenic, can cause various dysfunctions to plants, animals and humans. Therefore, their removal from polluted solutions becomes one of the focuses of environmental remediation. 

There are several physic-chemical methods to remove elements from wastewater such as adsorption, chemical precipitation, solvent extraction, reverse osmosis, ion exchange and chemical reduction [1,2,3]. Adsorption is recognized as an effective and economic method for the removal of metals and metalloids because it offers high efficiency and flexibility in operation [4]. 

Different materials with high specific surface areas such as activated carbons, resins and zeolites, have been widely used for wastewater treatment [2,5]. However, to minimize the cost of such materials and provide more efficiency for the removal of metals, alternative approaches have been developed using low cost materials such as agricultural waste by-products [2]. These include the use of modified clay [6,7], soil [8], seed powder [9], sugar cane bagasse [10], coffee and tea waste [11,12,13,14,15], neem bark [16] maize tassels [17], modified coconut fiber [18], coconut husk [19], rice husk [20], oil palm shell [21], fly ash, lime, agricultural ash and saw dust [22,23,24].

Among the low cost materials, food waste adsorbents compete favorably in terms of cost, efficiency and ease of operation [25]. Roughly one third of the food produced in the world for human consumption every year is wasted. Food waste amounts to roughly US $680 billion in industrialized countries and US $310 billion in developing countries. Fruits and vegetables, as well as roots and tubers have the highest wastage rates of any food [26]. Therefore, in the perspective of sustainable development, the identification of a possible alternative use of these food waste is important.

Recent studies have shown that banana peel [27], apple peel [28], eggplant peel [29], potato peel [30], orange peel [31], lemon peel [32,33], watermelon peel [34,35], tomato peel [36], coffee waste [11,15,37,38] decaf coffee waste, carob peel and grape waste [39,40] are efficient adsorbents for the removal of heavy metals and toxic elements from wastewater. However, the removal efficiency of food waste materials has been assessed by performing adsorption experiments in heterogeneous operating conditions. Consequently, it is difficult to compare the adsorption capacities of the individual food waste adsorbents. In fact, most existing studies are focused on the evaluation of the elements’ removal efficiency from synthetic mono-element solutions to define the adsorption isotherms and to obtain the optimum removal values of single elements. 

The aim of this study is to evaluate the adsorption capacities of 12 food waste materials (potato peel, lemon peel, orange peel, watermelon peel, tomato peel, coffee waste, apple peel, banana peel, decaf coffee waste, eggplant peel, carob peel and grape waste), comparing their efficiency for the removal of 23 elements (for the most of whom the adsorption capacities have never been assessed) from multi-element solutions (at pH 2.0 and pH 5.5), in homogeneous experimental conditions. 

The use of multi-element solutions does not allow the identification of the adsorption isotherms of the single elements and the determination of the interaction mechanism for the adsorption, which in part have already been defined in previous studies [11,15,27,28,29,30,31,32,33,34,35,36,37,38,39,40]. In fact, the competitiveness between the metals for the adsorbents’ active sites precludes obtaining the optimum removal values of the single elements. However, the use of multi-element solutions enables to evaluate and compare the efficiency of the individual food waste materials for the removal of various elements from a more complex matrix, closer to a real one. In fact, another goal of the study is to verify the potential of the food waste adsorbents on a real complex polluted matrix such as a heavy metal wastewater.

The porous surface of the food waste materials is particularly suited to adsorb different elements. In addition, the adsorbent surfaces have various types and amounts of functional groups, which may act as selective adsorption active sites for metals and metalloids. Other objectives of the study are to cluster the food waste materials through multivariate statistical computations of their FTIR spectra and to highlight possible correlations between the elements’ removal efficiency and the adsorbents’ chemical structures.

## 2. Materials and Methods

### 2.1. Preparation of the Food Waste Adsorbents

Potato peel, lemon peel, orange peel, watermelon peel, tomato peel, coffee waste, apple peel, banana peel, decaf coffee waste, eggplant peel, carob peel and grape waste were sun-dried for a week, grinded with a mortar and then sieved to retain particles sized between 0.25 and 0.125 mm. The obtained powders were washed three times with deionized water produced by an integrate water purification system (Arioso UP 900; Industrial Scientific Corporation, Pittsburg, PA, USA) and dried at 55 °C for 48 h in a vacutherm oven (Heraeus VT 6025; Kendro Laboratory Products, Hanau, Germany). Finally, the powders were weighed on an analytical balance (Gibertini Europe 60; Gibertini Elettronica Srl, Milano, Italy) to obtain different amounts of each adsorbent: 25, 50, 100 and 200 mg.

### 2.2 Preparation of Aqueous Solutions and Synthetic Multi-Element Solutions

Aqueous solutions at pH 2.0 and pH 5.5 were prepared using deionized water, HNO_3_ (LGC Promochem India Private Ltd, Bangalore, India) 1% and NaOH (Merck Millipore Ltd, Billerica, MA, USA) 5%. Synthetic multi-element solutions at pH 2.0 and pH 5.5, containing Ag, As, Ba, Cd, Ce, Co, Cr, Cu, Fe, Ga, In, La, Mo, Ni, Pb, Sb, Sn, Th, Ti, U, V, W, and Zn at the concentration of 1 mg/Kg were prepared by mixing different aliquots of mono-element standard solutions (Exaxol Italia Chemical Manufacturers Srl, Genoa, Italy; Ultra Scientific, North Kingstown, RI, USA; Merck Millipore Ltd, Billerica, MA, USA) into 10 mL of aqueous solution. The pH of the solutions was controlled using a pH meter (Crison MicropH 2002, Crisonb Instruments, Barcelona, Spain) and adjusted using HNO_3_ 1% and NaOH 5%. After 24 h, the synthetic multi-element solutions were filtered using syringes with cryolite transparent membranes (diameter: 25 mm, pore size: 0.45 µm; Merck Millipore Ltd, Billerica, MA, USA) to remove possible formed precipitates and then analyzed by inductively coupled plasma mass spectrometry (ICP-MS; Bruker 820-MS; Bruker Instruments, Billerica, MA, USA) to identify the elemental fractions really dissolved in solution (C_i_—initial concentration) before the adsorption processes.

### 2.3. Preparation of Wastewater

Wastewater produced in a hydro-metallurgical process for the recovery of valuable elements by electronic boards (WEEE—waste of electric and electronic equipment) was used to verify the efficiency of the 12 food waste adsorbents for the removal of heavy metals from a real polluted matrix. The wastewater was obtained after the acid leaching of electronic boards and the following removal of valuable elements through fractional precipitation of hydroxides. It is a very complex and variable matrix, and contains a high amount of salts as well as a high variable content of elements: Al < 100 µg/L; Ba = 318 µg/L; Cr = 146 µg/L; Cu = 377,000 µg/L; Fe < 200 µg/L; Mn = 7730 µg/L; Ni = 233,000 µg/L; Pb = 72,100 µg/L; Sn < 20 µg/L; and Zn = 2,650,000 µg/L [41]. Before performing adsorption experiments, the wastewater was diluted 1:100 with deionized water to reduce the amount of the adsorbates and to avoid the saturation of the adsorption active sites by the more concentrated metals. The pH of the diluted wastewater was 5.5.

### 2.4. Characterization of the Food Waste Adsorbents

The powders of the 12 food waste materials were analyzed by scanning electron microscopy (SEM; LEO 1450 VP; Carl Zeiss, Oberkochen, Germany) to assess their superficial aspect and porosity and by Fourier transform infrared spectroscopy (FTIR; IR Affinity Miracle 10; Shimadzu Scientific Instruments, Columbia, MD, USA) to identify the types and the amounts of functional groups present on their adsorbent surfaces. The powders were fixed to a specific support and coated with a thin layer of platinum metal before SEM analysis to make them surfaces conductive. The IR spectra were recorded in the range of 5000–600 cm^−1^ with a resolution of 5.0 cm^−1^.

### 2.5. Adsorption Experiments

Adsorption experiments were performed by treating each of the 12 food waste adsorbents with 10 mL of the synthetic multi-element solution (containing Ag, As, Ba, Cd, Ce, Co, Cr, Cu, Fe, Ga, In, La, Mo, Ni, Pb, Sb, Sn, Th, Ti, U, V, W, and Zn at the concentration of 1 ppm) under controlled pH conditions (at pH 2.0 and pH 5.5). Increasing amounts (25, 50, 100 and 200 mg) of each adsorbent were exposed, in different experiments, to the multi-element solutions (at pH 2.0 and pH 5.5). The pH of the solutions was controlled after the exposure of each adsorbent which may cause slight changes of pH. A final adsorption experiment was performed treating 200 mg of each of the 12 food waste adsorbents with 10 mL of the diluted wastewater (pH 5.5) produced in a hydro-metallurgical process. Before performing the adsorption experiments, solubility percentage of each element of the synthetic multi-element solution at different pH was verified (Figure S1).

At pH 2.0, elements’ solubility percentages are very high (except for W), thus at these acidic conditions it is possible to quantify the adsorption of the elements obtaining good quality analytical data. At pH 5.5, As, Ce, Fe, Ga, La, Pb, Sn, Th, Tl and U form insoluble hydroxides; consequently, their initial concentration (C_i_) is very low and it is not possible to accurately quantify their removal. Most of the considered elements completely precipitate at pH higher than 5.5; therefore, the adsorption experiments were not performed at basic conditions.

The aqueous solutions containing different food waste adsorbents (blanks) and the synthetic multi-element solutions with and without the adsorbents were magnetically stirred (New Variomag Electronicruhrer Telemodul; Thermo Fisher Scientific Inc., Waltham, MA, USA) at 25 ± 2 °C and 200 rpm for 24 h to ensure the achievement of the equilibrium between the adsorbates and the adsorbents. After 24 h, the samples were filtered using a glass filtering system with nitrocellulose membranes (diameter: 45 mm, pore size: 0.45 µm; Merck Millipore Ltd, Billerica, MA, USA), the pH of the solutions was controlled. All the adsorption experiments were performed in duplicate. 

Finally, samples were analyzed by inductively coupled plasma mass spectrometry (ICP-MS; Bruker 820-MS; Bruker Instruments, Billerica, MA, USA). Blank values due to the elements’ release from the adsorbent materials were also evaluated. Details about the used instrumental conditions are reported in Canepari et al. (2006) and Protano et al. (2016) [42,43]. 

### 2.6. Elements’ Adsorption Percentages

The elements’ adsorption percentages were calculated from the elemental fractions dissolved in the synthetic multi-element solution after 24 h from its preparation (after precipitation phenomena) and after its filtration (C_i_—initial concentration). In this way, the precipitated fractions of the elements, not available for the adsorption processes, were not considered as removed by the food waste materials.

For each food waste adsorbent, the elements’ adsorption percentages were calculated by subtracting to the concentration of the elements dissolved in solution before the adsorption processes (C_i_—initial concentration), the difference between the elemental fraction not removed by the food waste adsorbent (C_e_—equilibrium concentration) and the elemental fraction released in solution by the adsorbent itself (b_v_—blank value). This value was divided by the concentration of the element dissolved in the initial multi-element solution (C_i_—initial concentration) and multiplied per 100:

Adsorption % = [C_i_ − (C_e_ − b_v_)]/C_i_ × 100 (1)

### 2.7. Statistical Analyses

Multivariate statistical computations were performed using the statistical software R (R-project for statistical computing, Ver. 3.0, 32-bit). A first principal component analysis (PCA) was calculated on the spectral data obtained by FTIR spectroscopy of the food waste adsorbents in order to group the 12 materials as a function of the type and amount of functional groups present on their surfaces. A standard normal variate (SNV) autoscaling was operated to the spectral data before performing the PCA to correct multiplicative variations between the spectra caused by variations in sample physical properties or in sample preparation and presentation. A second PCA was performed on the data obtained by the adsorption experiments to group the food waste materials according to the obtained elements’ adsorption percentages.

## 3. Results and Discussion

### 3.1. SEM Micrographs

**Figure 1:**
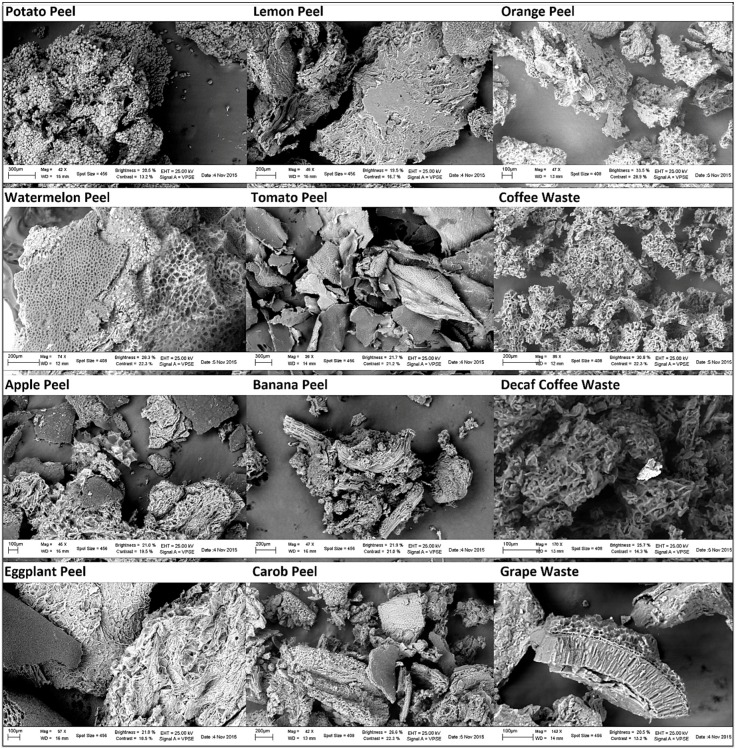
Adsorbent surfaces′ micrographs (at 100, 200 and 300 µm) obtained by scanning electron microscopy (SEM).

Different micrographs (Figure 1), which show the aspect and the porosity of the 12 adsorbent surfaces, were obtained by scanning electron microscopy (SEM). From the micrographs obtained by SEM, we observed that potato peel’s surface appeared composed by globular formations; tomato peel showed a layered and flat surface; banana peel and grape waste revealed fibrous and lamellar structures; and watermelon peel, coffee waste and decaf coffee waste possessed the most porous surfaces.

The 12 food waste materials showed various superficial structures characterized by peculiar conformations and different abundance, morphology and size of pores. Various grades of porosity correspond to different superficial specific areas available for adsorption processes. Therefore, from this first qualitative observation, we can suppose that the food waste materials with the most porous surfaces may be able to remove a higher amount of elements from polluted solutions.

### 3.2. FTIR Spectra

**Figure 2:**
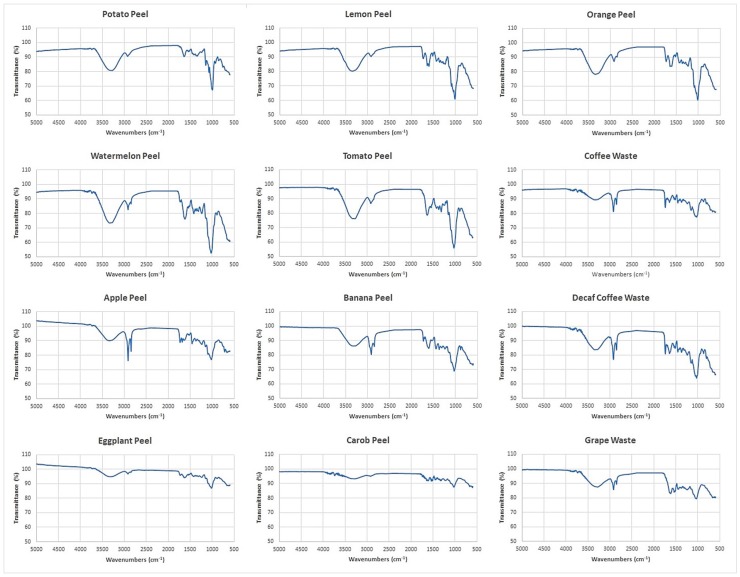
Adsorbent′s FTIR spectra obtained by Fourier transform infrared spectroscopy (FTIR).

By Fourier transform infrared (FTIR) spectroscopy, functional groups present on the adsorbent surfaces were identified. The FTIR spectra of the 12 food waste materials in the range of 5000–600 cm^−1^ are shown in Figure 2.

The broad band in the range of 3700 and 3000 cm^−1^ includes many vibrations modes corresponding to −OH of alcohols [44], phenols and carboxylic acids [36]. This region is greater in watermelon peel and tomato peel spectra, whereas it appears reduced in eggplant peel and carob peel spectra. The two sharp bands at 2925 cm^−1^ and 2855 cm^−1^ belong to the C−H bonds of methyl and methylene groups of lipids [44]. These bands are more intense in banana peel, apple peel, coffee waste and decaf coffee waste spectra. The band at 1742 cm^−1^ corresponds to the C=O vibration in the carbonyl group of −COOH. The bands in the range of 1600−1400 cm^−1^ can be ascribed to C=C vibration of lipids, fatty acids and lignin moieties [36,44,45,46,47]. Figure 2 shows that the bands from 1742 cm^−1^ to 1400 cm^−1^ are less intense in potato peel, carob peel and eggplant peel spectra. The region at 1400−900 cm^−1^ shows several types of vibrations including C−H, C−O−C, C−N and P−O of polysaccharides [44,48]. This region is greater in watermelon peel, tomato peel, lemon peel and orange peel spectra.

By the recorded FTIR spectra, similarities and differences of the chemical structures of the adsorbent surfaces were identified. Figure 2 reveals that each food waste material has different functional groups which may act as selective active sites for metal and metalloid ions coordination.

The spectral data obtained by FTIR spectroscopy were elaborated by performing multivariate statistical computations. Principal component analysis (PCA) of the spectral data allowed to group the food waste materials according to functional groups present on their adsorbent surfaces. 

**Figure 3:**
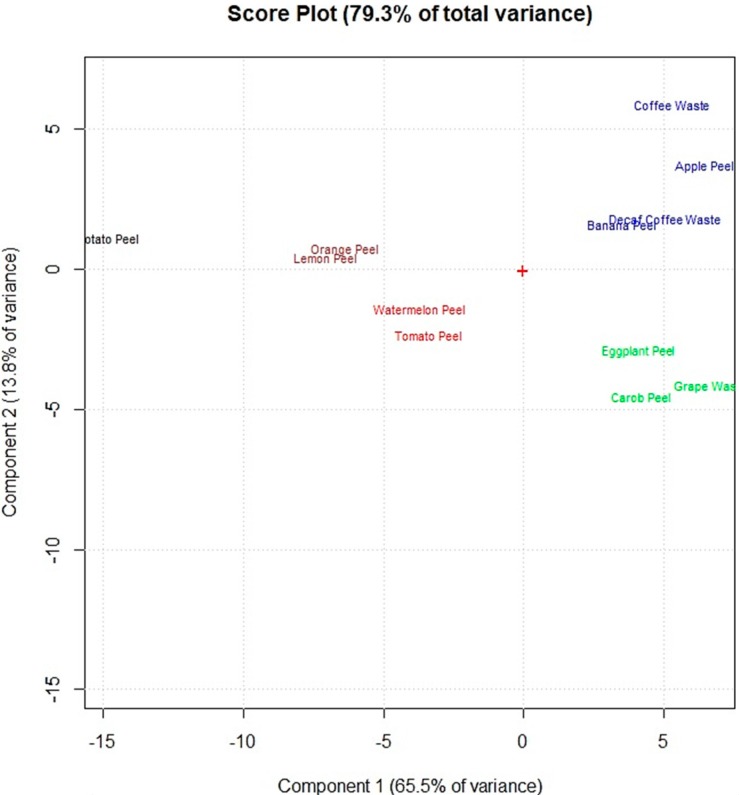
Score plot of the principal component analysis performed on the obtained FTIR spectral data of the 12 food waste adsorbents.

Figure 3 shows that the food waste adsorbents were grouped in five main clusters (marked in different colors) on the first two principal components (PC1 and PC2) of the score plot, which explain 75.7% of the total explored variance. A first cluster, composed by watermelon peel and tomato peel (red color) is placed on the bottom left part of the score plot’s center. The adsorbents of this cluster showed the highest amounts of −OH of alcohols, phenols and carboxylic acids; C−H, C−O−C, C−N and P−O of polysaccharides; and C=C of lipids and lignin moieties. Lemon peel and orange peel (brown color), which are placed on the upper left compared to the first cluster (red color), have a lower amount of these functional groups. Potato peel is away from lemon peel and orange peel mainly because of the lower amount of C−H, C−O−C, C−N, and P−O of polysaccharides and C=C of lipids and lignin moieties. The other two clusters are on the right of the score plot. The cluster above the score plot’ center (blue color) is composed by the adsorbents which showed the highest amount of C−H of methyl and methylene groups and lower amounts of the other functional groups: apple peel, banana peel, decaf coffee waste and coffee waste. The other one (green color), below the score plot’ center, is formed by eggplant peel, carob peel and grape waste which revealed the lowest amount of functional groups on their adsorbent surfaces.

### 3.3. Removal Efficiency of Food Waste Adsorbents from Synthetic Multi-Element Solutions

Food waste materials are efficient adsorbents for the removal of many metals and metalloids from synthetic multi-element solutions at pH 2.0 and pH 5.5. Table 1 shows that the adsorption capacity varies as a function of the pH and that each food waste adsorbent (200 mg) has a propensity to remove certain elements rather than others. At pH 5.5, As, Ce, Fe, Ga, La, Pb, Sn, Th, Tl, and U precipitate, thus it was not possible to quantify their removal. 

At pH 2.0, some elements, such as Mo, Pb, Sb, Sn, Th, Ti and W, were adsorbed in high percentages by most of the food waste materials while others were removed in different percentages depending on the adsorbent exposed. For example, As was removed only by banana peel (adsorption percentages ~37%) and by carob peel, which was able to adsorb more than 75% of the As dissolved in solution. At pH 5.5, the food waste materials resulted less selective for the adsorption of Ag, Cd, Mo, V and Zn, which were removed in high percentages by most of the adsorbents. Decaf coffee waste, coffee waste, potato peel, apple peel, eggplant peel and carob peel resulted more efficient at pH 5.5 than at pH 2.0 for the removal of Ba, Cd, Ni, V and Zn, whereas orange peel appeared more efficient at pH 2.0. W was removed in higher percentages at pH 2.0 by all the adsorbents except watermelon peel. Orange peel and tomato peel removed in higher percentages Ag and Cu at pH 2.0, while coffee waste, decaf coffee waste, apple peel, eggplant peel and carob peel appeared more efficient for the removal of these elements at pH 5.5. Finally, orange peel adsorbed more than 85% of Zn at pH 2.0, whereas apple peel, carob peel, coffee waste and decaf coffee waste were able to remove more than 85% of Zn only at pH 5.5.

Banana peel, watermelon peel and grape waste resulted the most efficient adsorbents for the removal of most of the metals and metalloids from multi-element solutions at pH 2.0 as well as at pH 5.5. Elements’ removal efficiency varies depending on the amount of adsorbent exposed in solution, as expected from the adsorption isotherms defined in previous studies [11,15,27,28,29,30,31,32,33,34,35,36,37,38,39,40].

**Figure 4:**
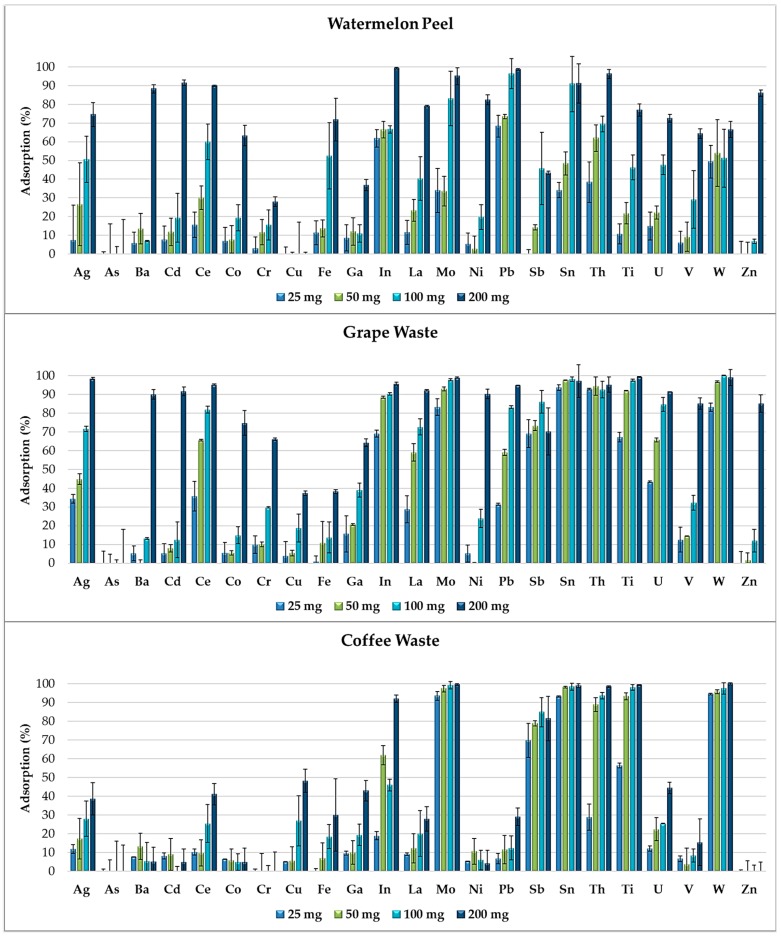
Removal efficiency of watermelon peel, grape waste and coffee waste, exposed in increasing amounts (25, 50, 100 and 200 mg) to the multi-element solution at pH 2.0.

Figure 4 shows that, by increasing the amount of adsorbent (25, 50, 100 and 200 mg) in the multi-element solutions, the adsorption capacity of the food waste materials increases and the selectivity for the elements’ removal decreases. In fact, metals and metalloids compete for certain active sites initially available in lower amounts on the adsorbent surfaces and completely occupied by the elements with the highest affinities. By doubling the amount of adsorbent, the removal percentages gradually increase because the higher availability of functional groups on the adsorbent surfaces progressively reduces the competitiveness between the elements.

In Figure 4, we observe that the adsorption percentages of some elements (such as Ag, Ce, Mo, La and Pb for grape waste and Ag, La, Mo, U and W for coffee waste) are strictly correlated with the amount of adsorbent exposed. Mo, Sb, Sn and W were removed in high percentages just by 25 mg of grape waste and by 25 mg of coffee waste. These elements favorably competed in the adsorption processes, demonstrating higher affinities to certain functional groups of such food waste materials. Instead Ba, Cd, Co, Ni and Zn were adsorbed in high percentages only by 200 mg of grape waste and watermelon peel which resulted the most efficient food waste adsorbents.

Standard deviations of the results obtained by the adsorption experiments (performed in duplicate) are all under 25%.

Each food waste material showed a propensity to remove certain elements rather than others depending on the functional groups present on its adsorbent surface.

**Figure 5:**
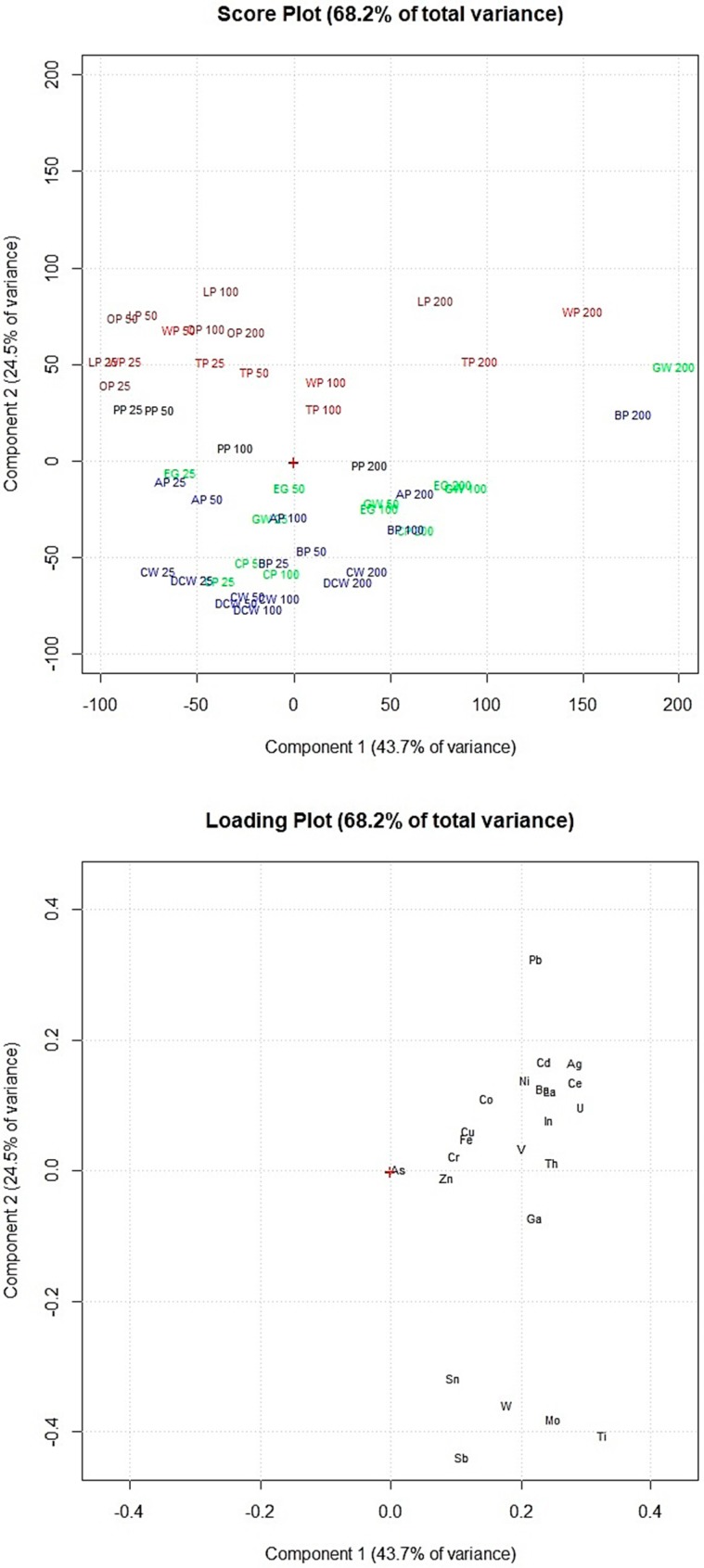
Score plot and loading plot of the PCA performed on the data obtained by the adsorption experiments at pH 2.0. PP, potato peel; LP, lemon peel; OP, orange peel; WP, watermelon peel; TP, tomato peel; CW, coffee waste; AP, apple peel; BP, banana peel; DCW, decaf coffee waste; EG, eggplant peel; CP, carob peel; GW, grape waste; 25, 25 mg of adsorbent; 50, 50 mg of adsorbent; 100, 100 mg of adsorbent; 200, 200 mg of adsorbent.

Principal component analysis of the data obtained by the adsorption experiments allowed clustering the food waste materials according to their elements’ adsorption percentages. Figure 5 shows the removal efficiency of the 12 food waste adsorbents, exposed in increasing amounts (25, 50, 100 and 200 mg) to the multi-element solution at pH 2.0.

Figure 5 shows that coffee waste and decaf coffee waste have similar elements’ removal efficiency as well as lemon peel and orange peel which are in the same part of the score plot. Sb, Sn, Mo, W and Ti (on the bottom part of the loading plot) were removed in high percentages by coffee waste and decaf coffee waste (on the bottom part of the score plot). Lemon peel, orange peel, watermelon peel and tomato peel (on the top of the score plot) showed lower adsorption percentages of these elements but appeared more efficient for the removal of the other metals and metalloids (Cr, Th, V, Fe, Cu, Co, In, U, Ba, La, Ni, Ce, Cd, Ag and Pb). Figure 5 shows that increasing the amount of adsorbent exposed, the elements’ removal efficiency considerably increased only for lemon peel, tomato peel, watermelon peel, grape waste and banana peel.

Comparing these results with the results obtained by the multivariate statistical analyses of the FTIR spectra allowed to highlight the potential correlations between the adsorbents’ efficiency and their specific chemical structures. The adsorbents in Figure 5 are marked in the same colors used in Figure 3 to graphically correlate the removal efficiency of the food waste materials with the chemical structures of the adsorbent surfaces. The adsorbents of the red and brown clusters (watermelon peel, tomato peel, lemon peel and orange peel), which showed the highest amounts of −OH of alcohols, phenols and carboxylic acids, of C−H, C−O−C, C−N and P−O of polysaccharides and of C=C of lipids and lignin moieties, resulted more efficient for the removal of Cr, Th, V, Fe, Cu, Co, In, U, Ba, La, Ni, Ce, Cd, Ag and Pb. Instead, the adsorbents of the blue and green clusters (banana peel, apple peel, coffee waste, decaf coffee waste, grape waste, eggplant peel and carob peel), which appeared with lower amounts of these functional groups, showed higher adsorption capacities of As, Zn, Ga, Sb, Sn, Mo, W and Ti. 

Therefore from the statistical elaboration of the obtained results we can suppose that the elements above the loading plot’s center of Figure 5 (Cr, Th, V, Fe, Cu, Co, In, U, Ba, La, Ni, Ce, Cd, Ag and Pb), which were removed in higher percentages by watermelon peel, tomato peel, orange peel and lemon peel, have higher affinities to −OH of alcohol groups and C−H, C−O−C, C−N and P−O of polysaccharides than the elements below the loading plot’s center (As, Zn, Ga, Sb, Sn, Mo, W and Ti).

### 3.4. Elements′ Removal Efficiency of Food Waste Adsorbents in Wastewater

The removal efficiency of the food waste adsorbents was verified on a real polluted matrix assessing the adsorption of heavy metals from the heavy metal wastewater produced in a hydro-metallurgical process. 

**Figure 6:**
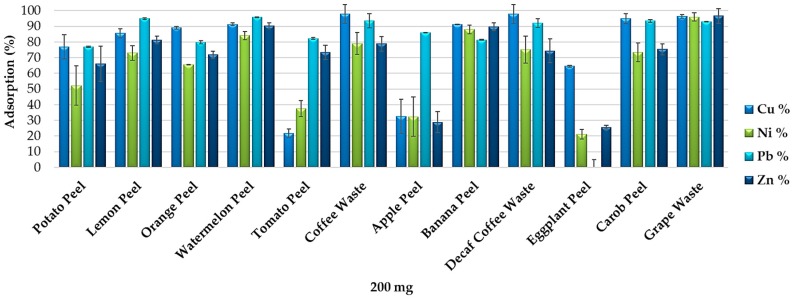
Removal efficiency of the 12 food waste adsorbents (200 mg) from the heavy metal wastewater produced in a hydro-metallurgical process (pH 5.5).

Figure 6 shows that the 12 food waste materials can be efficiently used for the removal of heavy metals from real wastewater. Each adsorbent showed different removal efficiency. Coffee waste and decaf coffee waste resulted the most efficient for the removal of Cu, watermelon peel for Pb and grape waste for Ni and Zn. Standard deviations of the results obtained by the adsorption experiments from the heavy metal wastewater (performed in duplicate) are under 20%.

These data confirm the results obtained by the adsorption experiments performed in synthetic multi-element solutions. However, food waste materials resulted less selective for the removal of metals and metalloids from the wastewater because of the lower competitiveness of the four heavy metals for the more available active sites of the adsorbent surfaces. On the other hand, banana peel, watermelon peel and grape waste resulted the most efficient and the least selective food waste adsorbents for the elements’ removal from the heavy metal wastewater as well as from the synthetic multi-element solutions. Although the use of multi-element solutions does not allow the individuation of the optimum elements’ removal values, it enables evaluating and comparing the efficiency of the individual food waste adsorbents for the removal of various elements from a matrix close to a real one.

## 4. Conclusions

Many food waste materials used as low cost adsorbent for the treatment of wastewater were reported in the past. This work was aimed to evaluate the adsorption capacities of most of the studied food waste materials comparing their efficiency for the removal of more than 20 elements from complex multi-element solutions, in homogeneous experimental conditions. 

The pH selected for adsorption experiments were pH 2.0 and pH 5.5. The latter pH value is just before the pH-zone of 5.5–8.0, where precipitation phenomena dominates. Elements’ removal efficiency was evaluated from complex multi-element solutions to assess the potential of the food waste materials in industrial applications.

Maintaining homogeneous experimental conditions offered the possibility to evaluate and compare the adsorption capacities of 12 food waste materials.

Banana peel, watermelon peel and grape waste resulted the most efficient and the least selective adsorbents for the removal of most of the metals and metalloids from multi-element solutions (at pH 2.0 and pH 5.5) as well as from heavy metal wastewater.

The adsorbent surfaces were analyzed by FTIR spectroscopy and showed different types and amounts of functional groups, which demonstrated to act as adsorption active sites for various elements. The 12 food waste materials were grouped, through multivariate statistical computations of the FTIR spectra, into five different clusters, depending on the functional groups present on their adsorbent surfaces.

By comparing these results with those obtained by the multivariate statistical analyses of the elements’ removal percentages, it was possible to highlight the potential correlations between the adsorbents’ efficiency and their specific chemical structures.

## Figures and Tables

**Table 1:** Removal efficiency of the food waste adsorbents (200 mg) from multi-element synthetic solutions at pH 2.0 and pH 5.5.

	Potato Peel	Lemon Peel	Orange Peel	Watermel. Peel	Tomato Peel	Coffee Waste	Apple Peel	Banana Peel	Decaf C. Waste	Eggplant Peel	Carob Peel	Grape Peel
	**Adsorption (%)**						**pH 2**					
	m ± SD	m ± SD	m ± SD	m ± SD	m ± SD	m ± SD	m ± SD	m ± SD	m ± SD	m ± SD	m ± SD	m ± SD
Ag	71 ± 8	96 ± 2	99 ± 5	75 ± 6	100 ± 1	39 ± 9	34 ± 24	94 ± 2	47 ± 8	70 ± 3	55 ± 3	98 ± 1
As	<10	<10	<10	<10	<10	<10	<10	37 ± 11	<10	<10	79 ± 2	<10
Ba	18 ± 12	56 ± 6	92 ± 1	88 ± 2	71 ± 5	<10	39 ± 17	80 ± 2	<10	14 ± 4	20 ± 4	90 ± 3
Cd	17 ± 11	64 ± 4	92 ± 1	91 ± 1	66 ± 4	<10	22 ± 5	88 ± 2	<10	15 ± 5	14 ± 4	92 ± 2
Ce	24 ± 3	68 ± 1	100 ± 1	90 ± 1	38 ± 2	41 ± 6	59 ± 7	77 ± 1	15 ± 6	72 ± 2	42 ± 3	95 ± 1
Co	<10	43 ± 6	43 ± 1	63 ± 5	32 ± 5	<10	14 ± 2	46 ± 5	<10	<10	<10	75 ± 7
Cr	<10	15 ± 7	20 ± 2	28 ± 3	47 ± 3	<10	34 ± 13	12 ± 2	14 ± 9	<10	43 ± 10	66 ± 1
Cu	51 ± 8	75 ± 3	75 ± 1	<10	62 ± 3	48 ± 6	49 ± 21	58 ± 1	43 ± 6	13 ± 1	60 ± 3	37 ± 1
Fe	42 ± 20	<10	<10	72 ± 11	38 ± 8	30 ± 19	13 ± 5	42 ± 2	11 ± 15	53 ± 13	40 ± 19	38 ± 1
Ga	31 ± 12	<10	<10	37 ± 3	29 ± 1	43 ± 5	40 ± 4	93 ± 1	41 ± 5	54 ± 4	36 ± 3	64 ± 2
In	73 ± 2	92 ± 1	76 ± 3	99 ± 1	55 ± 1	92 ± 2	92 ± 2	96 ± 1	87 ± 3	48 ± 1	89 ± 2	96 ± 1
La	15 ± 6	51 ± 1	75 ± 1	79 ± 1	37 ± 1	28 ± 7	40 ± 13	62 ± 1	33 ± 8	59 ± 1	22 ± 4	92 ± 1
Mo	94 ± 2	73 ± 2	87 ± 1	95 ± 5	88 ± 1	100 ± 1	99 ± 1	99 ± 1	100 ± 1	98 ± 1	99 ± 1	99 ± 1
Ni	<10	49 ± 5	87 ± 1	82 ± 3	36 ± 5	<10	<10	71 ± 3	<10	10 ± 3	10 ± 6	90 ± 3
Pb	77 ± 1	93 ± 1	<10	99 ± 1	90 ± 1	29 ± 5	88 ± 1	97 ± 1	17 ± 3	95 ± 5	79 ± 1	95 ± 1
Sb	46 ± 5	25 ± 5	19 ± 5	43 ± 1	32 ± 8	81 ± 12	82 ± 11	88 ± 14	83 ± 12	72 ± 15	93 ± 14	70 ± 13
Sn	86 ± 13	66 ± 26	57 ± 27	91 ± 10	59 ± 14	99 ± 1	92 ± 8	97 ± 1	99 ± 1	92 ± 1	99 ± 1	97 ± 9
Th	96 ± 1	96 ± 1	<10	96 ± 2	97 ± 1	98 ± 1	91 ± 5	98 ± 1	96 ± 1	97 ± 1	92 ± 1	95 ± 4
Ti	76 ± 6	61 ± 1	100 ± 1	77 ± 3	82 ± 1	99 ± 1	92 ± 4	99 ± 1	100 ± 1	98 ± 1	100 ± 1	99 ± 1
U	50 ± 7	45 ± 1	100 ± 1	72 ± 2	86 ± 1	44 ± 3	54 ± 7	88 ± 1	39 ± 3	82 ± 1	36 ± 4	91 ± 1
V	31 ± 12	13 ± 3	77 ± 1	64 ± 3	14 ± 9	15 ± 13	15 ± 1	75 ± 1	56 ± 9	26 ± 6	60 ± 3	85 ± 3
W	100 ± 5	67 ± 10	93 ± 14	67 ± 4	93 ± 3	100 ± 1	100 ± 2	100 ± 1	100 ± 1	100 ± 2	100 ± 1	99 ± 4
Zn	14 ± 12	55 ± 2	88 ± 2	86 ± 2	22 ± 5	<10	13 ± 7	72 ± 3	<10	<10	<10	85 ± 5
	**Adsorption (%)**						**pH 5.5**					
	m ± SD	m ± SD	m ± SD	m ± SD	m ± SD	m ± SD	m ± SD	m ± SD	m ± SD	m ± SD	m ± SD	m ± SD
Ag	94 ± 11	99 ± 3	<10	99 ± 14	29 ± 1	98 ± 13	99 ± 34	96 ± 7	99 ± 11	99 ± 6	100 ± 4	85 ± 2
Ba	89 ± 12	93 ± 7	<10	96 ± 2	97 ± 5	74 ± 13	99 ± 16	94 ± 10	98 ± 7	74 ± 8	92 ± 3	98 ± 2
Cd	90 ± 14	92 ± 12	12 ± 15	97 ± 11	79 ± 10	95 ± 7	95 ± 8	90 ± 8	95 ± 12	79 ± 8	94 ± 9	98 ± 9
Co	54 ± 10	45 ± 9	<10	41 ± 9	41 ± 6	56 ± 9	44 ± 2	37 ± 4	51 ± 8	38 ± 3	78 ± 4	48 ± 8
Cr	<10	15 ± 9	16 ± 10	23 ± 8	23 ± 5	<10	18 ± 11	30 ± 2	<10	29 ± 5	42 ± 9	11 ± 1
Cu	68 ± 18	78 ± 17	19 ± 5	92 ± 13	12 ± 8	95 ± 15	97 ± 28	77 ± 2	95 ± 9	85 ± 10	93 ± 8	86 ± 7
In	80 ± 4	72 ± 2	74 ± 5	86 ± 2	72 ± 1	66 ± 4	93 ± 2	98 ± 1	61 ± 4	52 ± 1	75 ± 5	96 ± 1
Mo	81 ± 5	80 ± 12	58 ± 7	94 ± 14	21 ± 2	93 ± 2	99 ± 10	95 ± 2	94 ± 1	97 ± 1	100 ± 2	74 ± 1
Ni	72 ± 11	85 ± 8	<10	94 ± 7	57 ± 8	93 ± 9	94 ± 12	88 ± 3	92 ± 11	65 ± 2	89 ± 6	96 ± 6
Sb	26 ± 4	25 ± 6	<10	32 ± 15	21 ± 8	52 ± 15	80 ± 16	86 ± 16	57 ± 14	52 ± 16	93 ± 15	<10
V	82 ± 9	64 ± 3	11 ± 7	90 ± 14	55 ± 7	90 ± 12	99 ± 3	95 ± 11	91 ± 10	97 ± 9	100 ± 5	95 ± 5
W	85 ± 9	90 ± 7	12 ± 19	95 ± 2	56 ± 10	90 ± 6	91 ± 8	79 ± 7	92 ± 15	37 ± 3	91 ± 9	96 ± 8
Zn	85 ± 9	90 ± 7	12 ± 19	95 ± 2	56 ± 10	96 ± 6	91 ± 8	79 ± 7	92 ± 15	37 ± 3	91 ± 9	96 ± 8

## Supplementary Materials

The following are available online at http://www.mdpi.com/1996-1944/11/3/334/s1. Figure S1: Removal efficiency of the 12 food waste adsorbents (200 mg) from the heavy metal wastewater produced in a hydro-metallurgical process (pH 5.5).
