# Supplementary material for: Efficiency Evaluation of Food Waste Materials for the Removal of Metals and Metalloids from Complex Multi-Element Solutions
Source: Materials (Basel). 2018 Feb 26;11(3):334. doi: 10.3390/ma11030334 (PMC5872913; doi:10.3390/ma11030334)
Supplement: Supplementary file 1 [file materials-11-00334-s001.pdf]

Supplementary

Before performing the adsorption experiments, solubility percentage of each element of the synthetic multi-element solution at different pH was verified. The solubility curves of all the elements were calculated at the experimental condition.

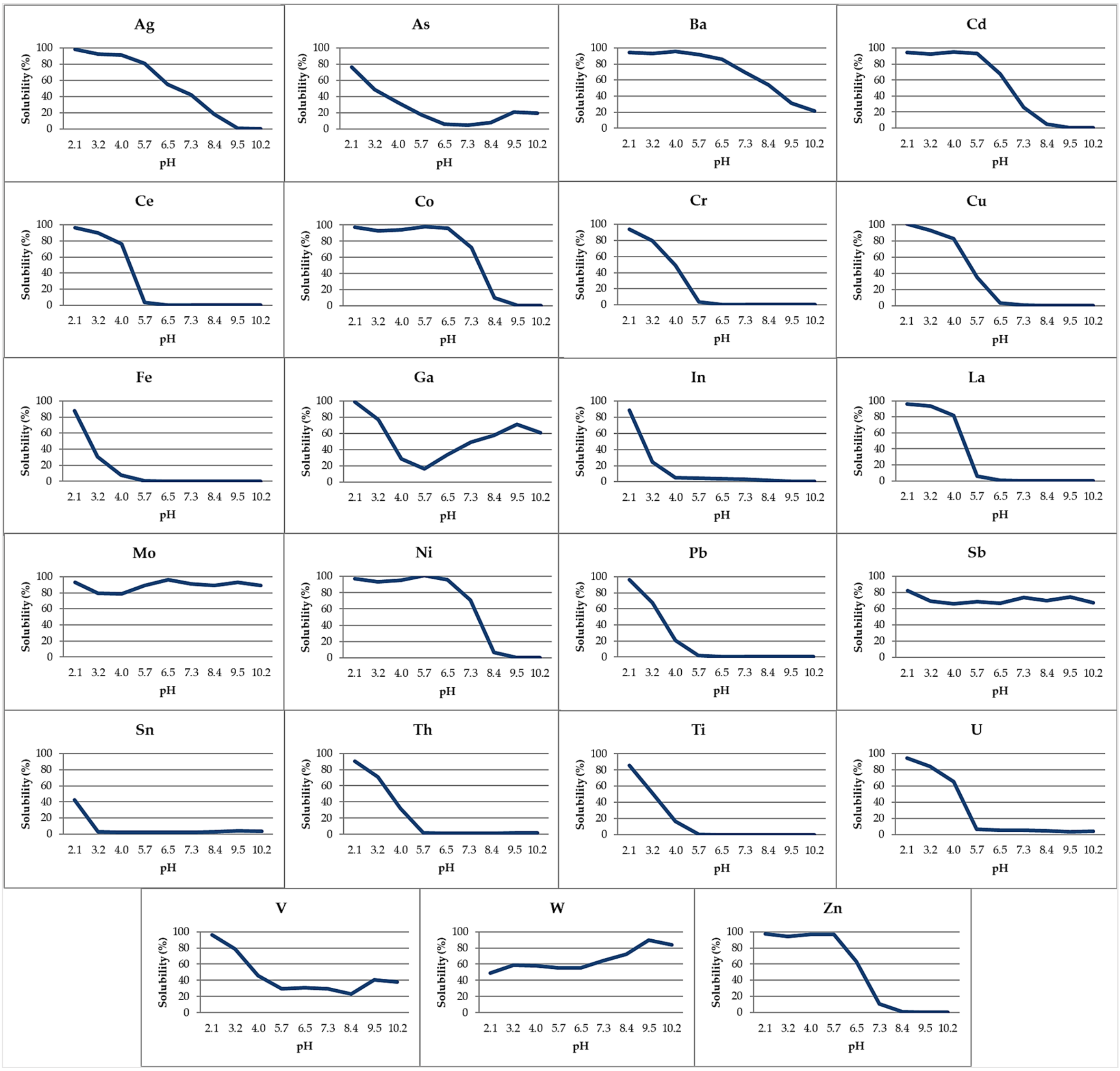

Figure S1. Removal efficiency of the 12 food waste adsorbents (200 mg) from the heavy metal wastewater produced in a hydro-metallurgical process (pH 5.5).

Synthetic multi-element solutions at different pH, containing Ag, As, Ba, Cd, Ce, Co, Cr, Cu, Fe, Ga, In, La, Mo, Ni, Pb, Sb, Sn, Th, Ti, U, V, W, Zn at the concentration of 1 mg/Kg were prepared by mixing different aliquots of mono-element standard solutions (Exaxol Italia Chemical Manufacturers Srl, Genoa, Italy; Ultra Scientific, North Kingstown, RI, USA; Merck Millipore Ltd, Billerica, MA, USA) into 10 mL of aqueous solution. The pH of the solutions was adjusted using HNO_3 (LGC Promochem India Private Ltd, Bangalore, India) 1% and NaOH (Merck Millipore Ltd, Billerica, MA, USA) 5% and controlled using a pH meter (Crison MicropH 2002; Crison Instruments, Barcelona, Spain). After 24 hours, the synthetic multi-element solutions were filtered using syringes with cryolite transparent membranes (diameter: 25 mm, pore size: 0.45 µm; Merck Millipore Ltd, Billerica, MA, USA) to remove possible formed precipitates and then analyzed by inductively

coupled plasma mass spectrometry (ICP-MS; Bruker 820-MS; Bruker Instruments, Billerica, MA, USA) to identify the elemental fractions really dissolved in solution (C_i – initial concentration) before the adsorption processes. From pH 2.1 to pH 4, elements' solubility percentages are very high (except for W), thus at these acidic conditions it is possible to quantify the adsorption of the elements obtaining good quality analytical data. From pH 5.7, As, Ce, Fe, Ga, La, Pb, Sn, Th, Tl and U form insoluble hydroxides; consequently, their initial concentration (Ci) is very low and it is not possible to accurately quantify their removal. Most of the considered elements completely precipitate at pH higher than 5.7; therefore, the adsorption experiments were not performed at basic conditions.

The elements' adsorption percentages were calculated from the elemental fractions dissolved in the synthetic multi-element solution after 24 hours from its preparation (after precipitation phenomena) and after its filtration (C_i – initial concentration). In this way the precipitated fractions of the elements, not available for the adsorption processes, were not considered as removed by the food waste materials.

© 2018 by the authors. Submitted for possible open access publication under the terms and conditions of the Creative Commons Attribution (CC BY) license (http://creativecommons.org/licenses/by/4.0/).
